# 100 years of crystallography: the IUCr launches a comprehensive open-access journal, **IUCrJ**

**DOI:** 10.1107/S2052252513033691

**Published:** 2013-12-19

**Authors:** S. Samar Hasnain

**Affiliations:** a Max Perutz Professor of Molecular Biophysics, University of Liverpool, Crown Street, Liverpool, Merseyside L69 7ZB, United Kingdom, and Editor-in-chief of IUCr Journals

**Keywords:** crystallography, editorial, IUCrJ

## Abstract

**IUCrJ** is a fully open-access journal that aims to publish high-quality structural science papers. It has been launched for the International Year of Crystallography (IYCr2014).

The year 2014 has been declared by the United Nations as the International Year of Crystallography (IYCr2014) to celebrate 100 years of success following the first Nobel prize related to crystallography, awarded in 1914 to Max von Laue. The International Union of Crystallography (IUCr), working with UNESCO, is proud to be the custodian of the IYCr2014 and has set up plans for major initiatives during the year. The Union has been working hard to prepare its journals (*Acta Crystallographica Sections A-F, Journal of Applied Crystallography* and *Journal of Synchrotron Radiation*) for the coming decades of crystallography-based science and for capturing developments in enabling technologies and methods.

The year coincides with the launch of a fully open-access journal with the objective of attracting high quality science papers of broad scientific significance from across all the scientific communities that use results obtained from diffraction methods. The journal is simply called **IUCrJ**, the first journal that carries the Union’s name in a manner similar to the well established journals PNAS and JACS that carry the names of the National Academy of Sciences (NAS) and the American Chemical Society (ACS).

Lawrence Bragg, who established the basis of X-ray crystallography, shared the Nobel Prize with his father William Bragg in 1915 at the age of 25. This second Nobel Prize related to crystallography was quickly followed a third, awarded to Charles Glover Barkla in 1917, who had established that the X-rays have the properties of light so that interference and diffraction processes should occur, as for light from a grating. Lawrence Bragg helped create the first international journal dedicated to crystallography, *Acta Crystallographica*, and worked to establish the IUCr as a member of the International Council for Science (ICSU). He was elected by the General Assembly of the first IUCr congress in 1948 as the first President with Max von Laue as the Honorary President. Paul Peter Ewald, who had also played a major role in the establishment of the IUCr, became the founding editor of *Acta Crystallographica* and remained its Editor until 1959. In the tradition established by Bragg, Laue and Ewald, we are pleased that many of the leaders of our hugely expanded and successful field have volunteered to serve on the editorial and advisory boards of **IUCrJ**, bringing together all of the expertise that only the Union can call upon. The journal has five main editors, three of whom are either current (Gautam Desiraju) or former (Ted Baker and Sine Larsen) Presidents of the Union.

Biologists, chemists, physicists and material scientists are encouraged to report the best of their structural studies in **IUCrJ**. Major scientific advances require multidisciplinary research and very often these breakthrough papers report results covering a wide range of methods and technologies. Our aim is to capture a fair share of high profile papers on all aspects of the sciences, technologies and methods supported by the IUCr through its commissions, including emerging fields such as three-dimensional structures from ‘single molecules’ using free-electron lasers (FELs). Many of the spectacular structural science results that are published in high profile journals appear first in presentations at IUCr congresses, and at AsCA, ECM and ACA meetings. The goal for 2014 will be to publish 100 articles (in line with the 100-year celebration of crystallography) in **IUCrJ** covering as many aspects of structural methods development and applications as possible. **IUCrJ** should become the natural home for reporting breakthroughs and ‘full’ science reports rather than simply a structure or how it was determined.

In addition to the sciences, **IUCrJ** will cover advances in technologies and methods. Sections on Synchrotron and Neutron Sources, led by Sine Larsen, and on Physics and FEL Science, led by John Spence, are aimed at capturing these advancing technologies as well as the frontier science that utilizes these advanced sources.

In keeping with our aims of securing a high impact for the journal, submissions undergo preliminary screening by a panel consisting of the five Main Editors (Ted Baker, Richard Catlow, Gautam Desiraju, Sine Larsen, John Spence) and the Editor-in-chief (Samar Hasnain). Three votes in favour will make a manuscript eligible for refereeing, and it will be immediately assigned to a Co-editor. This rigor at the submission stage is expected to provide a rapid and efficient review process benefiting both authors and reviewers.

The first issue of **IUCrJ** features papers from many of the areas we intend to cover including biology, chemistry, crystal engineering, materials, physics and FELs. We are grateful to these authors for submitting outstanding work that could have been published in many of the well established high-ranking journals. We look forward to receiving many of your papers that have wider scientific appeal. **IUCrJ** will publish original research articles in two forms with *Papers* extending to 7000 words and *Letters* limited to 4000 words. Both categories are designed to provide full accounts in a concise manner. The journal also welcomes *Topical Reviews* on any of the scientific areas that are covered by the Union.

